# Delineating Molecular Mechanisms of Squamous Tissue Homeostasis and Neoplasia: Focus on p63

**DOI:** 10.1155/2013/632028

**Published:** 2013-04-22

**Authors:** Kathryn E. King, Linan Ha, Tura Camilli, Wendy C. Weinberg

**Affiliations:** Division of Monoclonal Antibodies, Office of Biotechnology Products, Center for Drug Evaluation and Research, US Food and Drug Administration, Bethesda, MD 20892, USA

## Abstract

Mouse models have informed us that p63 is critical for normal epidermal development and homeostasis. The p53/p63/p73 family is expressed as multiple protein isoforms due to a combination of alternative promoter usage and C-terminal alternative splicing. These isoforms can mimic or interfere with one another, and their balance ultimately determines biological outcome in a context-dependent manner. While not frequently mutated, p63, and in particular the ΔNp63 subclass, is commonly overexpressed in human squamous cell cancers. *In vitro* keratinocytes and murine transgenic and transplantation models have been invaluable in elucidating the contribution of altered p63 levels to cancer development, and studies have identified the roles for ΔNp63 isoforms in keratinocyte survival and malignant progression, likely due in part to their transcriptional regulatory function. These findings can be extended to human cancers; for example, the novel recognition of NF**κ**B/c-Rel as a downstream effector of p63 has identified a role for NF**κ**B/c-Rel in human squamous cell cancers. These models will be critical in enhancing the understanding of the specific molecular mechanisms of cancer development and progression.

## 1. Introduction

p53 is a tumor suppressor that is upregulated and activated across organ systems as a tissue protective stress response mechanism [1]. *p63 *is a member of the *p53 *gene family which also includes *p73*. In contrast to p53, both p63 and p73 exhibit cell-type-specific expression patterns and exert tissue-specific functions [2, 3]. Relevant to this review, p63 plays an essential role in the development and maintenance of normal stratified squamous epithelium. All *p53* family members encode multiple protein isoforms that act in overlapping or opposing manners both within and across family members. Given the complexity of the p53 family and the potential for the different family members to mimic or interfere with each other, the balance of p53 family isoforms in a given cellular context can impact the biological outcome. In this review, we highlight how information derived from mouse models has provided insight into molecular mechanisms of normal keratinocyte growth regulation and human cancer pathogenesis. In particular, we focus on the *p63* gene, the role of its gene products in normal epidermal development and homeostasis, and how dysregulation of p63 protein expression, which is tightly controlled under normal conditions, contributes to squamous carcinogenesis, not only of the skin, but also in other squamous epithelial cancers such as those of the head and neck.

## 2. Overview of p63 Structure/Function 

Members of the p53 family were identified based on shared homology within their major functional domains: transactivation (TA), DNA binding (DBD), and oligomerization (OD); and exist as multiple protein isoforms due to a combination of alternate promoter usage and alternative splicing [4, 5]. Use of alternative promoters gives rise to isoforms of two classes: TA and ΔN. The TAp63 and TAp73 isoforms possess a transactivation domain with homology and function similar to that of p53, while the ΔNp63 and ΔNp73 isoforms lack this domain and can act to block TAp53-, TAp63-, and TAp73-mediated transcription [4] via the mechanisms discussed below. However, this does not imply that ΔNp63 isoforms lack transcriptional activation activity as alternate transactivation domains have been described both within the N-terminus of the ΔNp63 isoforms [6, 7] and in exons 11 and 12 of the C-terminus (transactivation domain 2 (TA2)) [8]. Further analysis has suggested that the second region is unlikely to be an independent activation domain [7]. Refined mapping studies indicate that this domain instead serves to modulate transcriptional activities associated with ΔNp63 isoforms [7]. Correspondingly, many positive transcriptional targets of ΔNp63 have been identified, which are discussed in this review.

**Figure 1:**
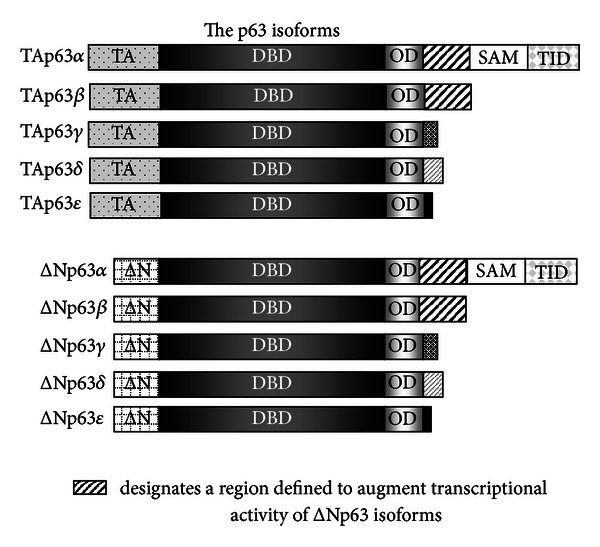
Use of alternative splicing gives rise to p63 isoforms of 2 subclasses: TAp63 and ΔNp63. Within each of these subclasses, C-terminal alternative splicing gives rise to *α*, *β*, *γ*, *δ*, and *ε* isoforms. The isoforms share homology in certain protein domains: TA (transactivation domain), ΔN, DBD (DNA binding domain), OD (oligomerization domain), SAM (sterile alpha motif domain), and TID (transactivation inhibition domain).

All TA and ΔNp63 isoforms contain the DBD and OD domains but differ at the C-termini. This additional complexity is conferred on these proteins due to C-terminal alternative splicing, which in the case of p63 gives rise to TA and ΔN subclasses of p63*α*, *β*, *γ*, *δ*, and *ε* isoforms [4, 9] (Figure 1). Of these isoforms, *α* is the longest and contains a sterile alpha motif (SAM) protein-protein interaction domain [10] and a transcriptional inhibition domain (TID) [11]. The TID comprises 2 subdomains, one of which binds and masks the TA domain of TAp63*α* and the other, which is subject to sumoylation resulting in decreased intracellular p63*α* concentration and correspondingly to decreased activity [11–13]. Degradation of p63*α* is also promoted by the E3 ubiquitin ligase ITCH via ubiquitylation at the N-terminal border of the SAM domain of the p63*α* isoforms [14]. Regarding the other p63 C-terminal splice variants, exon 13 is spliced out of the *β*-isoform, which thus also lacks the SAM and TID domains. Both the *α* and *β* isoforms of p63 contain a phosphodegron motif utilized by Fbw7 E3 ubiquitin ligase in MDM2-mediated degradation [15]. The *γ*, *δ*, and *ε* isoforms all truncate shortly after the oligomerization domain, with each containing a unique C-terminal sequence [9]. Thus, all three isoforms lack the SAM and TID domains (Figure 1).

 Like p53, the p63 and p73 proteins function as tetramers via their oligomerization domains. The oligomerization domains of p63 and p73, due to the presence of an additional *α*-helix, are more similar to one another than to that of p53 [16]. p63 and p73 were not observed to interact with p53 through their oligomerization domains but strongly interact with one another through this domain, with the p63/p73 heterotetramers exhibiting enhanced stability over homotetramers [16]. While p53 does not interact with p63/p73 through the oligomerization domain, WTp53 has been shown to target ΔNp63*α* for caspase-mediated degradation via interactions between the DNA binding domain of each protein [17], and mutated p53 has been shown to interact with the core DNA binding domains of p63 and p73, thereby impairing DNA binding and transactivation [18].

 DNA binding is an area in which p53 family members can mimic or compete with each other. While p63 has been shown to bind to p53 responsive consensus sequences, distinct p63 responsive elements have also been identified [19–21]. It has been reported that the global DNA binding pattern of p73 does not differ from that of p63, but intensity of binding at given sites does vary depending on the cell type profiled [22]. This suggests that competition between the homo- and heterotetramers of different isoforms of p63 and p73, which are subject to cellular context, may define site occupancy. Beyond interactions with one another via their oligomerization domains or their DNA binding domains, and their competition at DNA response elements, p53 family members have been shown to be involved in feedback loops with one another that impact expression levels [23, 24]. Thus, at many levels dysregultion of any one family member may impact the fine balance that is involved in maintaining normal epidermal homeostasis.

## 3. p63 and Normal Skin Biology

p63 is critical for normal epidermal morphogenesis [3, 25]. In the mature epidermis, the predominant p63 isoform expressed is ΔNp63*α*, and expression of this isoform is associated with the proliferative compartment [26]. Expression of this isoform is critical for the maintenance of the mature epidermis [27]. However, it is also the ΔNp63*α* isoform that is overexpressed in many squamous cell cancers [28]. As a starting point to understand how overexpression of a single isoform with ensuing disruption of the balance of p53 family members might contribute to squamous cancer pathogenesis, it is important to first understand the role of p63 both in normal epidermal morphogenesis and in homeostasis of the mature epidermis. Significant insight has been obtained through the use of mouse models outlined below.

### 3.1. Role of p63 in Mouse Models of Epidermal Morphogenesis and in Human Ectodermal Dysplasias 

#### 3.1.1. Mouse Models of Epidermal Morphogenesis

The criticality of p63 to normal epidermal development was highlighted by simultaneously published mouse models lacking functional *p63* [3, 25]. The mice were developed using distinct molecular strategies and upon gross phenotypic examination appeared similar; however, in-depth analysis of the epidermal phenotype gave rise to alternate hypotheses as to the role of p63 in epidermal development: epidermal progenitor cell maintenance versus commitment to stratification. In the mice developed by Yang et al., exons 6–8 corresponding to p63's DBD were replaced with the neomycin resistance gene [3]. In these mice, patches of disorganized epithelial cells positive for late markers of keratinocyte differentiation and negative for keratin 5 were evident, suggestive of a role for p63 in maintaining epidermal progenitor cells. Mice generated by Mills et al. were derived using an insertional gap repair mechanism [25]. Two strains generated by this approach, Brdm1 (truncating within exon 6) and Brdm2 (truncating after exon 10), appeared macroscopically identical, and thus, the strains were not distinguished in subsequent experiments in the seminal paper. Microscopic analysis of these mice revealed a layer of flattened cells expressing keratin 14 at low levels with no evidence of stratification or differentiation marker expression, suggestive of a failure to commit to a stratified epidermis. While no mRNA transcripts were detected from these mice by northern blotting, the transcript in the Brdm2 mouse model, which truncates after exon 10 [25], could in theory give rise to shortened ΔNp63 transcripts, similar to those described by Mangiulli et al. [9]. A recent recharacterization of a line of Brdm2 mice by Wolff et al. [29] revealed patches of keratinizing epidermis expressing truncated p63 at levels similar to wild type with stratification overlaying hair follicles. Based on further studies in embryos, the authors proposed these patches to be remnants of a more developed E15 epidermis 3–5 layers thick containing terminally differentiated epithelium that was transient in nature due to mechanical stress at birth, and suggested that the Brdm2 mice were equivalent to p63*α*/*β* knockout mice [29]. This observation and ensuing studies generated much controversy, as to whether the recharacterized mice were the same as those used by others or if perhaps a spontaneous genetic event might be at play [29–33], which to date remains unresolved.

Studies of the p63-deficient mouse lines provided strong evidence for the critical nature of the *p63* gene; however, interpretation of p63 function is confounded by the existence of multiple p63 protein isoforms. Therefore, single isoform knock-in mouse models have been developed on a *p63* null background to elucidate the role of specific p63 isoforms. These models have also generated controversy. Reconstitution of different p63 isoforms in the Brdm2 mice using tissue-specific inducible mouse models generated by separate groups gave rise to opposing conclusions as to the role of ΔNp63 in initiating stratification of simple epithelium [34, 35]. In one model, TAp63*α*, but not ΔNp63*α*, was found to drive stratification and keratin 5/keratin 14 expression of the simple lung epithelium [34], while in the other model, ΔNp63*α* or ΔNp63*β* caused stratification and keratin 5/keratin 14 expression in the simple lung epithelium [35]. With respect to the epidermis, differential results were also obtained by these two groups. In the first model, keratin 14-driven expression of TAp63*α* resulted in a severely hyperplastic epidermis exhibiting delayed differentiation [34], and based on their data the authors concluded that TAp63*α* is the initiating switch for epidermal stratification. In contrast, in the second model, expression of ΔNp63*α* or ΔNp63*β* under the control of the keratin 5 promoter did not result in complete restoration of epithelial integrity, but it did result in several areas of stratified epidermis, which expressed differentiation markers, indicating that the ΔNp63*α* and ΔNp63*β* can act to initiate stratification [35]. Further support for ΔNp63*α* as an initiator of stratification comes from other genetic complementation studies in which ΔNp63*α* or TAp63*α*, both under the keratin 5 promoter, was introduced into the *p63*(−/−) mice from Yang et al. [3, 36]. In these studies, ΔNp63*α* was able to partially restore the epidermal basal layer, but not differentiation marker expression, whereas TAp63*α* reconstitution resulted in a phenotype similar to *p63*(−/−) mice [36]. Reconstitution of a combination of ΔNp63*α* and TAp63*α* resulted in a more complete epidermis formation containing patches with a more organized structure that expressed markers of differentiation [36]. It is possible that differences with respect to the differentiation status of the epidermis generated by reconstitution of ΔNp63*α* in the later two studies could be due to the mouse model used, but in contrast to the first model discussed, partial epidermal restoration by ΔNp63*α* is a common feature of both.

Finally, subclass-specific knockout mice have been developed as a means of exploring functions attributable to the TAp63 or ΔNp63 subclasses in the presence of wild-type expression levels of the opposing subclass. Germline ablation of TAp63 did not impact normal epidermal morphogenesis in the presence of ΔNp63 isoforms [37]. In contrast, mice in which ΔN exon was replaced with GFP appeared phenotypically similar to *p63*(−/−) mice, and, like the *p63*(−/−) mice generated by Yang et al. [3], retained only disorganized patches of keratinocytes expressing terminal markers of differentiation [38]. However, in contrast to the mice generated by Yang et al. [3], these mice coexpressed keratin 5 along with the markers of terminal differentiation. Furthermore, expression of the basal transcription factor AP-2*α* indicated that in the absence of ΔNp63, basal patches can form, but these were observed to have decreased Ki67 staining. Taken together, this is suggestive of a role for ΔNp63 during epidermal morphogenesis in both progenitor cell maintenance and in epidermal commitment, closing the gap between the original interpretations of the pan-p63 mouse models [38]. 

#### 3.1.2. p63 and Human Genetic Syndromes

In humans, heterozygous mutations in *p63* are linked to genetic syndromes that include ectodermal dysplasia as part of the disease phenotype [39]. Distinct phenotypes are associated with mutations in specific p63 domains, providing clues to structure/function relationships. For example, ankyloblepharon-ectodermal defects-cleft lip/palate (AEC) syndrome is associated with mutations in the SAM domain [40], while ectodermal dysplasia and cleft/lip palate (EEC) syndrome is associated with mutations within the DBD [41, 42]. Not all of the syndromes include skin involvement. Of the syndromes, skin involvement is most pronounced and severe in AEC, which is linked to missense mutations in the SAM domain [40, 43] and therefore implicates the *α* isoforms.

Unlike normal skin where ΔNp63 expression is associated with the basal proliferative compartment, in AEC patient skin samples, nuclear p63 expression extends beyond the basal layer to the terminally differentiating cells and is accompanied by coexpression of differentiation markers [40]. *In vitro* studies have shown that SAM domain mutations, as seen in AEC, block interaction between p63*α* and mRNA splicing/processing proteins critical to direct splicing of FGFR-2 to the isoform required for normal epithelial differentiation [44]. Characterization of the AEC L514F ΔNp63*α* mutant in stable cell lines revealed that activation of a cryptic splice site due to loss of these interactions resulted in production of a C-terminally truncated form of ΔNp63*α* exclusively located in the nucleus and resistant to proteosome degradation [45]. Mouse models have helped to further define the contribution of this genetic alteration to the phenotype of this syndrome. A knock-in mouse model developed for the L514F mutation recapitulates the expected AEC phenotype including hypoplastic and fragile skin with a transient reduction in proliferation during embryonic development [46]. Skin fragility in these mice, and in humans with AEC syndrome, was associated with altered desmosome gene expression mediated by mutant p63 [47]. To gain insight into further pathways impacted, intact and eroded AEC syndrome skin and normal skin were compared by microarray analysis. The findings revealed changes in expression of genes associated with epidermal adhesion, skin barrier formation, and hair follicle biology, all consistent with the clinical presentation [48]. Thus, SAM domain mutations highlight the importance of p63 and in particular ΔNp63*α* to normal epidermal morphogenesis/homeostasis. 

### 3.2. p63 in Normal Epidermal Homeostasis

**Figure 2:**
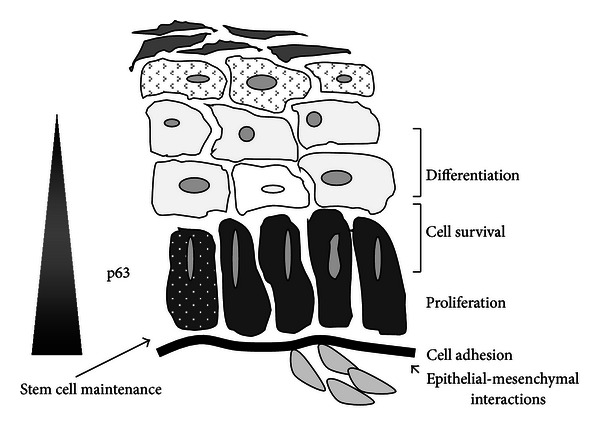
p63 impacts multiple biological endpoints involved in normal epidermal homeostasis. Overexpression of ΔNp63*α* impacts pathways that can contribute to cancer development.

Maintenance of normal epidermal homeostasis involves mediation of processes including proliferation, differentiation, stem cell maintenance, senescence, viability, and cell adhesion. Evidence suggests that each of these is impacted by p63 protein expression (Figure 2). *In vivo,* in the adult human epidermis, p63 is highly expressed in the basal cells with proliferative potential [26] and is downregulated in the suprabasal layers [4]. *In vitro* depletion of p63 in human regenerating organotypic cultures resulted in hypoproliferation and a lack of stratification and differentiation [49]. These effects were found to be mainly due to the ΔNp63*α* isoform. In a mouse model, specific knockdown of ΔNp63*α* in the mature epidermis resulted in severe skin fragility with erosion [27]. A multitude of studies, primarily *in vitro*, focused on the downstream targets mediated by ΔNp63*α* have shed light on the network of target genes implicated in these ΔNp63*α*-mediated biological processes. While an extensive cataloguing of all of these studies is beyond the scope of this review, some of these studies, with a focus on those performed in keratinocytes, are discussed here to highlight the potential impact of dysregulated ΔNp63*α* on signaling pathways that may be assessed using mouse models of the skin.

#### 3.2.1. Cell Cycle Regulation

Numerous examples serve to illustrate how perturbation of ΔNp63 expression could result in altered biological outcome. *In vitro*, in developmentally mature murine keratinocytes, we and others demonstrated that ΔNp63*α* is associated with maintenance of proliferative capacity [49–53]. Mimicking overexpression of ΔNp63*α* seen in squamous cell carcinomas blocks the normal growth arrest and induction of the cyclin-dependent kinase inhibitor p21^WAF1^ in response to elevated Ca^2+^ conditions [50, 51] and correspondingly suppresses the differentiation markers keratin 10 and filaggrin, but not keratin 1. The *α*-tail of ΔNp63 is required for its suppressive effect on differentiation but not for the aberrant growth arrest response [51], which may be mediated at least in part by transcriptional repression of p21^WAF1^ by binding of ΔNp63*α* to its promoter [52]. Regulation of p21^WAF1^ is further impacted by crosstalk between p63 and Notch 1, whereby Notch 1 is negatively regulated by ΔNp63*α* in cells of high renewal potential but synergizes with ΔNp63*α* during early differentiation to induce keratin 1. Subsequently, Notch 1 downregulates ΔNp63*α* to permit the expression of the late differentiation marker involucrin [54]. This context-dependent crosstalk is implicated in maintaining the balance between keratinocyte growth arrest and differentiation. 

In addition to the factors regulating p21^WAF1^ described above, a balance exists between ΔNp63*α* and many other target genes involved in proliferation and differentiation that are critical for maintenance of or for the switch between the states. For example, the cell cycle inhibitor PTEN is negatively regulated by ΔNp63*α*. Depletion of either ΔNp63*α* or PTEN alone had opposite effects on colony growth in colony forming assays, but depletion of both ΔNp63*α* and PTEN at the same time had no impact, implying the balance between the two is critical to biological outcome [55]. Another example focuses on the epidermis of mice with mutant IRF6, which is hyperproliferative and fails to undergo differentiation [56]. This has been attributed to a failure of a feedback loop with ΔNp63*α* that controls ΔNp63*α* expression, thereby regulating the switch between proliferation and differentiation. In this feedback loop, IRF6 is a direct transcriptional target of ΔNp63*α*, which when upregulated induced proteasome-mediated degradation of ΔNp63*α* allowing for keratinocytes to exit the cell cycle [57]. In addition to regulation of levels of ΔNp63*α* impacting biological outcome as exemplified by the previous two examples, Runx1, a transcription factor involved both in keratinocyte proliferation and differentiation, is directly differentially regulated by ΔNp63*α* in proliferating versus differentiating keratinocytes by binding to distinct DNA binding sites on the Runx1 promoter. This represents a different mechanism of regulation [58], however, one that also could be perturbed by altered expression of ΔNp63*α*. 

#### 3.2.2. Differentiation

The mouse models described in Section 3.1.1 support a role for ΔNp63*α* not only in the maintenance of epidermal progenitor cells, but also, in the commitment to stratification. At a molecular level, ΔNp63*α* synergizes with Notch 1 to induce keratin 1 expression during differentiation, and the ΔNp63*α* target gene *IKK*α** is necessary for epidermal differentiation [54, 59–63]. However, overexpression of ΔNp63*α* in primary murine keratinocyte cultures blocks expression of keratin 10 and filaggrin, but not keratin 1 [50] implying that a fine balance in levels of ΔNp63*α* is required for complete differentiation. Some examples of transcription factors which based on *in vitro* studies are thought to interplay with ΔNp63*α* during differentiation follow. 

Basally expressed keratin 14 is a known direct transcriptional target of ΔNp63*α* [64]. The transcription factor Skn1a (Oct11) blocks ΔNp63*α* induction of the keratin 14 promoter and promotes keratin 10 upregulation [65]. There is also evidence for ΔNp63*α* both blocking and inducing transcription factors that promote differentiation in a cell-context-dependent manner. For instance, ΔNp63*α* directly represses high-mobility group box protein 1 (HBP1), a transcription factor necessary for stratification of organotypic cultures [66]. In contrast, in differentiating keratinocytes ΔNp63*α* induces ZNF750, a transcription factor that is required for terminal epidermal differentiation [67]. Interestingly, ZNF750 is bound by ΔNp63*α* in both proliferating and differentiating keratinocytes, but expression is only induced in differentiating cells, suggesting that additional cofactors are involved for distinct biological endpoints. The contribution of cofactors is further exemplified by the case of Alox12, a granular layer protein important for epidermal barrier formation, which is induced by ΔNp63*α* only in differentiating keratinocytes [68]. 

ΔNp63*α* expression can be regulated at the transcriptional level as well as by altered protein stability, as noted above. Another means of controlling levels of ΔNp63*α* is by microRNAs, short RNA molecules that act as posttranscriptional regulators. They recognize seed sequences in the 3′UTR and serve to block protein translation or decrease RNA stability. Such an interaction was identified as part of a feedback loop between p63 and iASPP, an inhibitory member of the apoptosis stimulating protein of p53 family, critical for epidermal homeostasis [69]. In this loop, iASPP is a direct transcriptional target of p63 that positively regulates ΔNp63 via the repression of miRs 754-3p and 720 to allow for proliferation. Blocking iASPP expression allows for differentiation via upregulation of miRs 754-3p and 720, which downregulate ΔNp63*α*. Other examples include miR203, which directly targets p63 through its 3′UTR for degradation and promotes differentiation by restricting proliferative potential and promoting cell cycle exit [70]. miRs are also regulated by ΔNp63*α*. miR-34a and miR-34c, associated with cell cycle withdrawal, are negatively regulated by ΔNp63*α* [71]. In contrast, miR17, miR20b, miR30a, miR106a, miR143, and miR 455-3p are positively regulated by p63 and critical for the onset of keratinocyte differentiation via modulation of the MAPKs [72]. 

#### 3.2.3. Epidermal-Dermal Interface and Adhesiveness and Viability

Adhesiveness and cell viability are two additional properties positively impacted by ΔNp63*α*. Epidermal-specific knockdown of ΔNp63 in mature keratinocytes in mice resulted in impaired differentiation and compromised basement membranes [63]. In an *in vitro* model, *Fras1*, which encodes for an extracellular matrix protein, was identified as a ΔNp63*α* regulated gene important for maintaining the epidermal-dermal interface integrity [63]. To maintain this interface, p63 prevents nonepidermal gene expression in keratinocytes via positive regulation of bone morphogenetic protein- (BMP-) 7 [73]. The importance of ΔNp63 in maintaining epithelial-mesenchymal crosstalk was highlighted by the discovery of *interleukin-1 α* *(Il-1*α*)* as a p63 target gene. IL-1*α* induces growth factors in fibroblasts that can bind to receptors on the basal keratinocytes to promote proliferation [74]. Cell-cell adhesiveness was found to be mediated by p63 via Perp, which is a critical desmosomal component for cell-cell adhesion in normal development and in wound healing [75, 76]. With respect to apoptosis, the proapoptotic protein, IGFBP3, is directly repressed by ΔNp63 in both normal and SCC cells [77]. Similarly, downregulation of p63 in primary human foreskin keratinocytes was found to induce apoptosis and to reduce both *β*1 and *β*4 integrin expression [78], linking adhesiveness with viability. 

Taken together, the data presented in Sections 3.2.1–3.2.3 demonstrate that the network of genes regulated by ΔNp63*α* is large and perturbation of the balance between ΔNp63*α* and members of this network could have adverse biological consequences. 

### 3.3. Stem Cell Maintenance and Senescence

The proliferative lifespan of cells is limited by replicative senescence during which the cells permanently withdraw from the cell cycle, yet remain viable [79, 80]. This phenomenon is associated with the normal ageing process of renewable tissues such as the epidermis. p63 has been proposed as a marker of human epidermal keratinocyte stem cells that is downregulated when keratinocytes become transient amplifying cells [81]. Consistent with these data and the hypothesis that epidermal progenitor cell exhaustion occurs in *p63*(−/−) mice, depletion of p63 in immature human epidermal keratinocytes resulted in reduced clonal growth [82]. Regulation of replicative senescence in human epidermal keratinocytes involves miRs-138, 181a, 181b, and 130b which promote senescence by targeting ΔNp63*α* and Sirt1 for degradation. However, in a feedback loop, these miRs are themselves targets of negative regulation by ΔNp63*α* [83]; thus, overexpression of ΔNp63*α* could also perturb senescence.

Mouse models provide support for a role for p63 in the maintenance of stem cell proliferative capacity. Characteristics of accelerating ageing were noted in *p63*(+/−) mice observed for extended periods generated by two groups using the mice developed by both Yang et al. and Mills et al. [3, 25, 84, 85]. Germline or somatic *p63* depletion under control of the keratin 5 promoter gave rise to enhanced senescence marker expression [84], suggesting a role for p63 in the negative regulation of senescence. Indeed, overexpression of ΔNp63*α* in primary mouse keratinocytes overcame replicative senescence in association with delayed and diminished induction of INK4/p16 and Arf/p19 [86]. Consistent with these findings, crossing of *p63*(−/−) mice developed by Yang et al. [3] with *INK4/p16*(−/−) or *Arf/p19*(−/−) mice was able to partially rescue the proliferation and differentiation defects observed in *p63*(−/−) mice [87], reinforcing a role for p63 in blocking senescence. While these mice display reepithelialization, skin from *p63*(−/−) mice crossed with *Ink4a/p16*(−/−) or *Arf/p19*(−/−) mice is fragile and easily detachable, suggestive of defective adhesion, which also can be attributed to p63. 

In the mouse models described above, all p63 isoforms were knocked down. A TAp63-specific knockdown mouse model that supports a role for TAp63 in adult stem cell maintenance was generated by crossing TAp63 floxed mice with germline-specific promoter cre or keratin 14-cre mice [37]. *TAp63*(−/−) mice exhibited signs of premature ageing. Interestingly, overexpression of ΔNp63*α* under control of the keratin 14 promoter resulted in a phenotype similar to that reported in mice lacking TAp63 [88]. Skin-derived precursor (SKP) cells are multipotent precursor cells derived from the dermis that can differentiate into mesodermal and neural cells [89]. In the *TAp63*(−/−) mice, SKP cells proliferate more rapidly than wild-type SKP cells, and thus, undergo senescence more rapidly. As adult stem cell populations are not immortal, this enhanced proliferation in *TAp63*(−/−) cells would be expected to lead to stem cell exhaustion, which is associated with accelerated ageing. 

## 4. p63 and Neoplasia

### 4.1. Observational Studies of Human Tumors

The *p53* tumor suppressor gene is commonly mutated in human cancer [1]. Due to the similarity of the TAp63 isoforms with p53, it was hypothesized that mutation of p*63* could provide a mechanistic explanation for tumors in which *p53 *was not mutated. It was found, instead, that mutation of *p63* is a rare event in human cancer cell lines [90], but that p63 overexpression is seen in human squamous cell cancers including esophageal squamous cell carcinoma [91, 92], nasopharyngeal carcinoma [93], and squamous cell carcinoma of the skin [94, 95]. Overexpression of the ΔNp63 protein in primary squamous cell carcinomas (SCCs) of the head, neck, and lung correlates with amplification of the p63 gene locus, which occurs frequently in these cancers [28, 96]. While there is agreement that ΔNp63*α* is overexpressed in lung SCCs, conflicting results have been published as to whether this correlates with prognosis [96, 97]. 

In squamous cell carcinomas of the skin, a significant increase in p63 expression, both in terms of intensity and distribution, is seen relative to normal skin, as the proliferative fraction is expanded in tumors [26, 95]. Examination of skin lesions ranging from keratoacanthoma to a grade IV spindle cell carcinoma revealed very strong p63 immunoreactivity in grade 3 SCC with decrease in a single grade IV spindle SCC. In these tumors, carcinoma *in situ* was characterized by p63 immunoreactivity in all layers [94]. While ΔNp63*α* was shown to be the most overexpressed isoform in squamous cell tumors, careful characterization of the TA and ΔN isoforms from different tissue and tumor types revealed that individual isoforms are differentially expressed in the neoplastic transformation of different tissue types [98], implying specific contributions of the isoform expressed in a context-dependent manner. While ΔNp63*α* is overexpressed in primary skin tumors, expression of TAp63 is not a common event but has been reported to be downregulated relative to normal skin using PCR-based methods [99]. It is clear that ΔNp63*α* is overexpressed in skin SCCs, however, whether it actively plays a role in tumor formation or is a bystander has been unclear. Further insight into this question has been gained by *in vitro* and *in vivo* studies, as discussed below.

### 4.2. *In Vitro*/Molecular Studies with Human Cancer Cell Lines and Primary Keratinocytes

#### 4.2.1. Impact on Signaling Pathways

In particular, *in vitro* studies in SCC cells have provided insight into the potential signaling pathways impacted by p63 dysregulation in squamous cell carcinoma. As discussed previously, IRF6 is involved in a negative feedback loop with ΔNp63*α* that is necessary for the downregulation of ΔNp63*α* seen with differentiation [57], and an appropriate balance between these factors is required for the switch between proliferation and differentiation in the normal epidermis [100]. Correspondingly, expression of IRF6 was found to be strongly downregulated in human SCC [101]. Reexpression of IRF-6 in the context of primary human keratinocytes expressing both ΔNp63*α* and a mutant v-ras 12 oncogene was found to abolish the ability of ΔNp63*α* to promote colony growth and restore oncogene induced senescence [101], supporting a role for IRF6 in regulating ΔNp63*α* as part of its tumor suppressor function. In other studies, ΔNp63*α* has been shown to upregulate Hsp70, a protein colocalized with ΔNp63 in primary SCCs of the head and neck (HNSCCs) that is associated with proliferation and viability of HNSCC [102]. Likewise, accumulation of *β*-catenin in the nucleus and activation of downstream signaling pathways common to many cancers are induced by ΔNp63*α* in HNSCC cells [103].

Consistent with a role in promoting adhesion, ΔNp63 is negatively regulated by the epithelial-to-mesenchymal transition (EMT) promoting transcription factors snail and slug, and this association is observed in primary human cervical, head and neck, and esophageal SCCs. This decrease in ΔNp63*α* is associated with increased migration in SCC cell lines [104]. ΔNp63*α* also physically sequesters YB-1, a positive translational mediator of snail, thereby preventing both enhanced snail activity and YB1's function in actin cytoskeleton reorganization, both of which lead to cancer cell migration and invasion [105]. Another direct transcriptional target of ΔNp63*α* is the vitamin D receptor (VDR) [106], which is induced by multiple p63 isoforms. Downregulation of VDR expression results in increased cell migration of A431 epidermoid carcinoma cells, which can be rescued by ΔNp63*α* or VDR [107]. A role for ΔNp63*α* in preventing metastasis is further supported by the finding that antagonism of ΔNp63*α* by mutant-p53/Smad complex allows TGF-*β* to convert from a tumor suppressor role to a role in promoting metastases [108]. In line with this, knockdown of p63 in squamous cancer cell lines, in which the predominant isoform expressed was ΔNp63, led to an increase of mesenchymal and neural markers and upregulation of genes associated with invasion and motility [109]. 

Based on the data, it is enticing to contemplate that ΔNp63*α* plays a role in cancer development by promoting proliferation and viability at earlier stages, while it may need to be downregulated during progression to allow for the necessary enhanced motility, invasiveness, and EMT [110] that allow metastases to form. 

#### 4.2.2. Altered Responsiveness to Genotoxic Stress

ΔNp63*α* can impact cellular response to genotoxic stress. A mouse model in which ΔNp63 was overexpressed under control of the loricrin promoter showed that downregulation of ΔNp63 is required for UVB-induced apoptosis of the epidermis [111]. Mechanistically, degradation of ΔNp63*α* in keratinocytes exposed to apoptotic doses of UV was shown to be mediated by p38 MAPK, which phosphorylates ΔNp63*α*. This led to its detachment from p53-dependent promoters and results in apoptosis induction [112]. Consistent with this report, occupancy of binding sites involved in cell cycle arrest and apoptosis switched following adriamycin or UV treatment of human epidermal keratinocytes from ΔNp63*α* to p53 occupancy, which would be expected to result in increased apoptosis or cell cycle arrest [113]. 

Many therapeutic agents used in cancer treatment promote genotoxic stress as a means to reduce or control tumor growth. Expression of high levels of ΔNp63*α* predicts responsiveness of primary HNSCC to platinum-based therapies [114]. Upon exposure to cisplatin, ΔNp63*α* is proteosomally degraded via stratifin-mediated nuclear export and Rack1 targeting [114, 115]. An interaction between the p63 proteins and the NF-*κ*B pathway also plays a role in responsiveness to chemotherapeutics. In JHU-022 oral cavity SCC cells, IKK*β*, a known activator of RelA, promotes ΔNp63*α* degradation in response to cisplatin [116]. In this cell line, cisplatin treatment resulted in a physical interaction between RelA and ΔNp63*α* that abrogates ΔNp63*α* mediated p21^WAF1^ promoter repression and targets ΔNp63*α* for proteosomal degradation [117]. The presence of c-Abl, which has been implicated as an oncogene, in HNSCC cells treated with cisplatin stabilizes ΔNp63*α* expression. This stabilization of ΔNp63*α* leads to enhanced cell viability [118], which could be anticipated to result in clinical consequences. 

Survival of HNSCC cells that overexpress ΔNp63*α* is dependent on the presence of ΔNp63*α*, which functions by blocking TAp73-driven apoptosis both via promoter binding and physical interaction with p73 in a p53-independent manner [119]. TAp73 and ΔNp63*α* are engaged in a feedback loop involving miR-193a-5p, which is repressed by ΔNp63*α* and activated by TAp73 and targets the p73 UTR. Cisplatin treatment results in ΔNp63*α* degradation and TAp73-mediated activation of miR-193a-5p, limiting TAp73's pro-apoptotic effects and chemosensitivity [23]. Reimplantation in the presence or absence of a miR-193a-5p antagomir of disaggregated cells from primary mouse SCCs generated by a chemical carcinogenesis protocol revealed that knockdown of this miR resulted in reduced tumor formation and enhanced chemosensitivity [23], indicating that a strategy targeting both ΔNp63*α* and miR-193a-5p might be more effective in this scenario. HNSCC cells can circumvent the requirement for ΔNp63*α* expression for survival by the overexpression of Bcl2 [119]. In addition to blocking p73 to promote survival of HNSCC, ΔNp63*α* associates with histone deacetylase 1 and 2 forming an apoptotic transcriptional repressor complex. This complex is sensitive to breakdown by cisplatin and HDAC inhibitors, in the presence of low, but not high, levels of endogenous Bcl-2 indicating once again that the context of the tumor impacts the success of chemotherapy [120]. Unlike the case of TAp73 described above, in HaCaT cells in response to chemotherapy, ΔNp63*α* is involved in an antiapoptotic feedback loop in which it, as well as mutant p53, induces ΔNp63*α* [24]. Thus, response to genotoxic stress is another biological endpoint that can be impacted by dysregulated ΔNp63*α*.

### 4.3. Modeling Human Cancers in Mouse to Assess the Contribution of p63 to Neoplasia

Cancer arises as a multistep process that can be reiterated in well-established mouse models in a controlled fashion [121]. Results of the studies presented above suggest that TAp63 would harbor tumor suppressor properties and overexpressed ΔNp63 would harbor oncogenic properties. In this section, we highlight the use of mouse models to dissect out how altered p63 levels contribute biologically to prevention or development of cancer, either alone or in altered balance with other family members or other oncogenic pathways. Approaches discussed utilize mice with a heterozygous null mutation in *p63* on a background of wild type, *p53 *(+/−) and/or *p73 *(+/−)*;* TA-isoform-specific knockout mice; and mouse models where the elevated levels of ΔNp63*α* observed in human SCC are mimicked in cultured keratinocytes and transplanted to nude mice. 

#### 4.3.1. TAp63 as a Tumor Suppressor

The potential role for physiological levels of p63 acting as a tumor suppressor with respect to spontaneous tumor development was explored by two groups in the context of alteration of other p53 family members [85, 122]. In a mouse model in which the *p63* genotype was contributed by mice developed by Yang et al. [3], mice heterozygous for a null mutation in both *p63* and *p73* displayed a higher incidence of spontaneous tumor formation relative to wild-type mice. Furthermore, mice heterozygous for *p53*,* p63*, and *p73* developed a higher incidence and formed more aggressive tumors than mice heterozygous for the *p53 *null mutation alone. These findings suggest that p63 and p73 share a tumor suppressor role as has been long established for p53 [1, 85]. In the absence of additional genetic mutations, these *p63*(+/−) mice developed squamous cell carcinomas (10%), adenomas (15%), and histiocytic sarcomas (20%) at 10%, 15%, and 20% greater rates, respectively, than wild type. In contrast, in a study using mice with a p63 genotype contributed by the mice developed by Mills et al. [25], *p63*(+/−)/*p53*(+/−) mice were found to be less prone to spontaneous tumors than p53 +/− mice alone. Additionally, these *p63*(+/−) mice were shown to have decreased susceptibility to chemically induced carcinogenesis, suggesting that p63 does not contribute a tumor suppressor activity in cancer. To date, this controversy remains unresolved.

In the mouse models described above, all p63 isoforms were targeted. TAp63-subclass-specific knockdown mice allow distinction between the TA and ΔN subclass properties. Following observation for 2.5 years, an enhanced incidence of carcinoma, including SCC of the skin and sarcoma development, was observed in *TAp63*(+/−) and *TAp63*(−/−) mice relative to wild-type mice [123], again supporting a tumor suppressive role for TAp63. It was noted that tumors from the *TAp63*(+/−) and *TAp63*(−/−) mice were highly metastatic, and at a mechanistic level TAp63 was found to positively regulate Dicer, a protein critical for miR processing, and miR 130b. Reexpression of both Dicer and miR130b in *TAp63*(−/−) MEFs decreased invasiveness of these cells, suggesting that TAp63's tumor suppressor role could be mediated at least in part through Dicer and miR130b [123]. As mentioned previously, miR130b targets ΔNp63*α* for degradation [83].

#### 4.3.2. Overexpressed ΔNp63*α* Facilitates Tumor Progression

The mouse models described above focused on the TAp63 isoforms and were performed at wild-type or decreased levels of endogenous p63. However, overexpression of ΔNp63*α* is a common event in squamous cancers. Two independent studies have used similar approaches to mimic this overexpression with the goal of examining the *in vivo* functional consequences of ΔNp63*α* overexpression in the epidermis. Results from both lab groups support a contributory role for ΔNp63*α* in the cancer phenotype with mechanistic distinctions. In studies performed in our laboratory, wildtype primary murine keratinocytes were transduced with retrovirus encoding a v-rasHA oncogene in combination with a lentivirus encoding either a control GFP construct or ΔNp63*α* and grafted onto the dorsum of nude mice in combination with primary dermal fibroblasts [86]. This model allows growth of normal keratinocytes as well as benign and malignant tumor phenotypes in the graft site. Mice were observed up to a month following cell grafting for tumor formation. No lesions were observed in graft sites following transplantation of keratinocytes expressing only GFP or ΔNp63*α* alone. Grafting of keratinocytes expressing v-Ras^HA^ + GFP resulted, as expected, in the formation of well differentiated papillomas, while grafting of keratinocytes expressing v-Ras^HA^ + ΔNp63*α* resulted in 100% malignant conversion to carcinoma [86]. Although elevated levels of ΔNp63*α* alone are insufficient to confer a tumor phenotype *in vivo*, we found that ΔNp63*α* blocks oncogene-induced senescence by inhibiting p16^ink4a^/p19^arf^ pathways and cooperates with oncogenic v-Ras^HA^ to enhance malignant conversion *in vivo*. This study supports a contributory role for ΔNp63*α* in cancer pathogenesis and a mechanistic link to cell survival by overriding oncogene-induced senescence through inhibition of p16^ink4a^ and p19^arf^, key mediators of cellular senescence. 

Using a similar approach, Keyes et al. [124] demonstrated that overexpressing ΔNp63*α* in keratinocytes in the presence of oncogenic ras resulted in growth of malignant carcinomas following subcutaneous injection. In this study also, the malignant phenotype was associated with a bypass of oncogene-induced senescence. Overexpression of ΔNp63*α* was further shown to enhance stem-like proliferation of keratinocytes and maintain survival of the keratin 15-positive stem cell population. Furthermore, chromatin-remodeling protein Lsh was identified as a new target of ΔNp63*α* and as an essential mediator of senescence bypass. Although p19^arf^ was not detectable in the tumors derived from ras/ΔNp63*α* keratinocytes in this study, an *in vitro* component of the study indicated that p16^ink4a^ and p19^arf^ were not reduced during the initial stages of senescence bypass. Therefore, contrary to our study, it was proposed that the initiating events through which ΔNp63*α* inhibits senescence do not occur via p16^ink4a^/p19^arf^ pathways. Although the difference in p16^ink4a^/p19^arf^ between these two studies may be due to the different time courses used, it further indicates the complexity of the pathways interacting with p63 family members and underscores the need for additional studies to understand the role of p63 and its downstream effectors in tumorigenesis and senescence.

An oncogenic role for ΔNp63 is further supported by studies in a mouse model containing a dominant negative 14-3-3*σ* mutation (*Er/+*). 14-3-3*σ*, a protein associated with keratinocyte differentiation, is a direct target for ΔNp63*α* repression in undifferentiated human epidermal keratinocytes [52]. Treatment of *Er/+* mice on a *p63*(*+/+*) background with a two-stage carcinogenesis protocol resulted in the formation of tumors in which ΔNp63*α* was strongly expressed, while loss of function of an endogenous allele of p63 in this context, which generated (*Er/+/p63 *+/−) mice, resulted in reduced sensitivity to this protocol, suggestive of cooperation of ΔNp63*α* in Ras/14-3-3*σ*-induced tumorigenesis [125]. 

*A Role for NF-*κ*B/c-Rel in ΔNp63*α*-Mediated Carcinogenesis. *In a transcription factor profiling exercise, we identified activation of NF-*κ*B in keratinocytes following the overexpression of ΔNp63*α*. The NF-*κ*B family comprises 5 members functioning as hetero- and homodimers [126]. Only NF-*κ*B/c-Rel was found to be modulated by ΔNp63*α* under these conditions, with nuclear accumulation of phosphorylated c-Rel but none of the other NF*κ*B subunits enhanced in the presence of overexpressed ΔNp63*α*. NF-*κ*B is associated with multiple human diseases, including cancer, for which therapeutics targeting its constitutive NF-*κ*B activation are under development [127, 128]. Of the five family members, RelA, p50, and c-Rel subunits have been implicated in the maintenance of normal epidermal homeostasis [129, 130], and in SCC, the RelA/p50 heterodimer has been shown to promote or repress malignancy in a context-dependent manner [131, 132]. Nuclear c-Rel expression is associated with both solid breast tumors and hematopoietic malignancies [133]; however, it had not previously been investigated in SCC of the skin. The increase in nuclear c-Rel accumulation seen with elevated ΔNp63*α* levels was found to be critical to the ability of ΔNp63*α* overexpressing keratinocytes to proliferate under conditions that normally induce growth arrest [134]. Mechanistically, this is correlated with a physical interaction between ΔNp63*α* and c-Rel on the promoter of the p21^WAF1^ gene in these cells, both *in vitro* and *in vivo*, which represses p21^WAF1^ expression. These findings extended to primary human HNSCC, in which we found that ΔNp63*α* and c-Rel colocalized in the nuclei throughout the tumor sections, as opposed to a more restricted expression in normal tissue. 

In an extension of these studies, Lu et al. characterized a dynamic mechanism whereby c-Rel, ΔNp63*α*, and TAp73, which are coexpressed in the nuclei of a subset of HNSCC cell lines, control expression on binding sites including p21^WAF1^, Noxa, and Puma [135]. Exposure of HNSCC cell line cultures to TNF-*α* to mimic inflammation in the tumor environment was found to induce nuclear accumulation of c-Rel. In the absence of this stimulus, ΔNp63*α* was found to physically interact with TAp73. Similar to our results in untreated primary keratinocytes upon overexpression of ΔNp63*α*, a physical interaction between endogenous ΔNp63*α* and c-Rel was observed following TNF-*α* treatment in these HNSCC cell lines. Interestingly, under conditions of c-Rel overexpression, the interaction between ΔNp63*α* and TAp73 was blocked and TAp73 was translocated to the cytoplasm. This suggests that c-Rel displaces TAp73. ChIP assays indicated that this regulation occurred on the promoters of genes involved in growth arrest and apoptosis, resulting in their downregulation [135]. 

## 5. Summary/Future Directions: Challenging *In Vitro* Findings in *In Vivo Models*

Primary murine cell cultures and *in vivo *murine models have been instrumental in elucidating the multistep nature of carcinogenesis [121] and in challenging the role of specific genetic alterations, such as those observed in *p63*, in cancer pathogenesis [86, 124]. The mouse models described in this review have provided us with a clear picture of the importance of p63 to normal epidermal development and homeostasis and have highlighted the roles for specific p63 isoforms in neoplasia. However, given the complexity of the p63 family members, their interactions, and the context-specific manner in which they can exert their effects, much remains to be defined. 

Mouse models with molecular alterations that allow targeting of specific gene products will be indispensible in deepening our understanding of and resolving controversy related to the role of *p63* both in normal tissue and in disease. Once a pathway has been implicated, primary cultures from mouse epidermis can be readily manipulated to express or eliminate a particular protein presumed active upstream or downstream to assess the impact. Applied in combination with keratinocytes from genetically altered mice, both *in vitro* and *in vivo* findings can be challenged further for the consequences of the alterations. However, it is important to remember that all models have limitations, and a deeper understanding of the role of p63 in normal epidermal homeostasis and neoplasia will ultimately be derived from an iterative process involving *in vitro* observations in primary cells, cell lines and primary tumors, *in vivo* queries of these findings, and reexamination of the outcomes in the context of human tumors. Mouse models comprise a critical component of this process. 

As an example, the observation in primary mouse keratinocytes that c-Rel acts downstream of ΔNp63*α* in modulating keratinocyte growth regulation led to a further novel observation that c-Rel levels are enhanced in primary HNSCC of humans and links this protein accumulation to altered NF*κ*B/c-Rel activity in human head and neck squamous cell cancer cells [134]. The requirement for c-Rel in these cancers can be tested by modulating c-Rel and ΔNp63*α* independently using lentiviral gene transduction followed by grafting. Long-term overexpression of ΔNp63*α* has been shown to support sustained high levels of nuclear c-Rel expression, and c-Rel shRNA lentiviruses are capable of depleting c-Rel in keratinocytes for extended time (unpublished observations). Assessing the impact of these modulations *in vivo* will clarify the interplay between these alterations and their relevance to cancer development and progression.
